# Anger management in substance abuse based on cognitive behavioral therapy: an interventional study

**DOI:** 10.1186/s12888-017-1511-z

**Published:** 2017-11-23

**Authors:** Ladan Zarshenas, Mehdi Baneshi, Farkhondeh Sharif, Ebrahim Moghimi Sarani

**Affiliations:** 1 Department of Mental Health and Psychiatric Nursing, Community Based Nursing and Midwifery Research Center, School of Nursing and Midwifery, Shiraz University of Medical Sciences, Shiraz, Iran; 2 Department of Nursing, School of Nursing and Midwifery, Student Research Committee, Shiraz University of Medical Sciences, Shiraz, Iran; 3 Community Based Psychiatric Care Research Center, Shiraz University of Medical Sciences, Shiraz, Iran; 4 Department of Psychiatry, Research Center for Psychiatry and Behavioural Science, Shiraz University of Medical Sciences, Shiraz, Iran

**Keywords:** Aggression, Anger management, Cognitive behavioral therapy, Substances abuse

## Abstract

**Background:**

Anger and aggression have been developing notably in societies, especially among patients depending on substance abuse. Therefore, this study aimed to investigate the effect of anger management based on group education among patients depending on substances according to Patrick Reilly’s cognitive behavioral approach.

**Methods:**

In a quasi- experimental study, all patients who met the inclusion criteria were evaluated regarding their aggression level. The participants were assigned to 12 educational sessions based on group therapy and Patrick-Reilly’s anger management by focusing on using a combination of cognitive intervention, relaxation, and communication skills. The data were analyzed using the SPSS statistical software, version 16.

**Results:**

The findings showed a significant difference between the two groups regarding aggression level after the intervention (*p* = 0.001). No significant relationship was observed between aggression level and demographic variables (*p* > 0.05).

**Conclusion:**

The intervention of this study can be used for establishing self-management and decreasing anger among patients depending on substances. They can also be used as a therapeutic program in addition to pharmacotherapy.

**Trial registration:**

IRCT2016102030398N1.

## Background

Anger arousal is often known as an adaptive response to affective discomfort, which is represented by aggressive behaviors [1] and can affect human relations. It is in fact a feeling observed before aggressive behaviors. Studies have shown that individuals with high levels of anger got involved in verbal and physical aggression [2]. This might lead to aggressive behaviors towards family members and other individuals [3]. The prevalence of aggression in a study by Nooripour et al., [4] was reported to be 5% to 20%. Substance abuse and its consequences are among the most common health problems around the world [5]. Therapists often believe that anger and aggression are associated with substance abuse. Investigations have also shown that 40% of cocaine consumers suffered from different levels of aggression [6]. According to the World Health Organization, although law enforcement due to fear of punishment can have an impact on the behavior, change in beliefs that reduce aggression and crime requires more time and interventions [7]. Such methods led to reverse results in some cases compared to interventional programs based on penalty and positive reinforcement among prisoners in a British camp. The results of that study indicated that the rate of crime recommitment was increased by focusing more on penalty [8]. Research found that anger management has a positive effect on the prevention of offender behavior [9]. In fact, benefitting from anger management skills led to an increase in individuals’ adjustment ability as well as psychological capability [10]. Cognitive behavioral therapy is a method used for treating a large number of mental disorders. It basically focuses on recognizing incorrect, negative, and illogical beliefs affecting patients’ affection, behaviors, and belief reconstruction [11]. The Patrick-Reilly approach is a cognitive behavioral approach based on a combination of cognitive interventions, relaxation, and communication skills. During therapeutic sessions based on Patrick-Reilly’s approach, different strategies are provided for controlling anger initiation and its consistency. In addition, some tasks are given to participants in order to guarantee learning. A number of these strategies include relaxation through respiration, progressive muscle relaxation, thought blocking, and assertion skills [6]. Considering the importance of anger and aggression control among patients abusing substances, researcher’s experience about the frequency of aggression and physical conflicts between patients and nurses in psychiatric wards, and families’ concern about management of this misbehavior after their discharge, the present study aims to perform anger management group education based on Patrick Reilly’s cognitive behavioral therapy approach. The aim of the intervention is to provide participants with education according to evidence-based and scientific findings in ways that help them to gradually be able to control and manage their anger through simple strategies and skills.

## Methods

### Study design

This study was a non-randomized trial with pretest/posttest evaluation on patients divided into an intervention and a control group.

### Sampling

The study population included all patients admitted in Ebnesina Hospital, Shiraz, Iran. Considering α = 0.05 and power of 80% and using NCCS software, a 40-subject sample size was determined for the study (20 subjects in each group). The effect size based on the previous study was calculated as about 1 (mean different = 23, SD = 14).

The inclusion criteria of the study were depending on substances and not suffering from any psychiatric disorders affecting aggression (such as schizophrenia, bipolar disorder, PTSD and psychotic disorder that was diagnosed by the psychiatrist) On the other hand, the exclusion criteria were abusing substances and not participating in educational sessions. “Depending on substances” vs. “abusing substances” was differentiated by the psychiatrist according to the DSM-IV-TR criteria. In addition, the users of amphetamines and stimulants who were suffering from psychotic symptoms and could not participate in group therapy were excluded from the research sample.

**Fig. 1:**
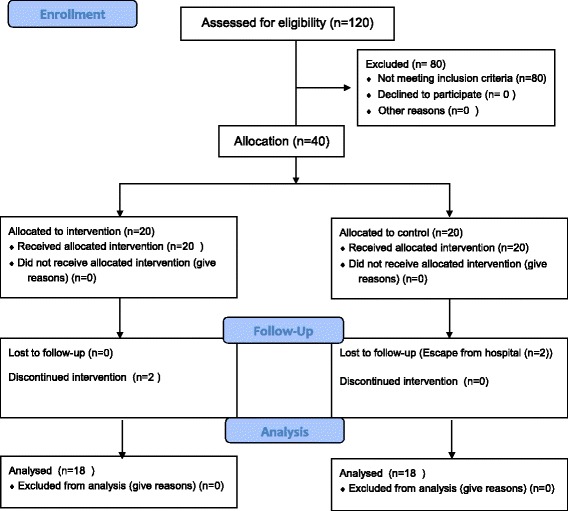
Flow chart of study design

First, patients who were admitted in the Ebnesina Hospital and met the inclusion criteria were selected (*n* = 120). Then, Aggression Questionnaire (AGQ) was used to measure their aggression levels. Those with a high aggression level were identified. Of these, 20 were non-randomly assigned to the intervention group and 20 in the control group (Fig. 1). The members of the two groups were not able to communicate with each other during the intervention. Although the staff were not aware which patients were in either of the groups, and also the patients did not communicate with each other and share information since they had been selected from different parts, but complete blinding was not possible because of the intervention was performed by the researcher in the intervention group.It should be mentioned that of the 40 participants, 2 patients in the intervention group refused to attend the treatment sessions and also 2 patients in the control group fled from the hospital. Since the doors of psychiatric hospitals are closed in Iran and the patients are not able to leave the hospital freely, most of patients with mental illness who are addicted to drug abuse are not willing to stay in the hospital.

### Ethical considerations

This study was approved by the Research Vice-chancellor and Ethics Committee of Shiraz University of Medical Sciences (95–10,537).

### Procedure

First, the study objectives and procedures were explained to the participants and their informed consents were obtained. Then, the participants were non-random divided into an intervention and a control group. The first group received an educational anger management course based on Patrick Reilly’s cognitive behavioral therapy. The treatment sessions were held in the hospital ward. In doing so, they were classified into 10-group units and received 12 1.5 h educational sessions. The content of these sessions consisted of anger management, anger recognition, cognitive behavioral strategies for anger control, assistant techniques for anger control, and impact of pervious learning and practical tasks. Meanwhile, routine education was provided for the control group participants. One week after finishing the course, the rate of aggression was measured by AGQ in both study groups.

### Statistic methods

Data analysis was performed using SPSS software (Version 16). To determine the participants’ demographic characteristics, descriptive statistics including absolute and relative frequency distribution, mean, and standard deviation were used. In addition, intergroup comparisons were performed using independent samples t-test. To evaluate the effectiveness of the treatment in each group, paired samples t-test and chi-square test were used. Finally, to compare the difference between the two groups in terms of more than three variables, One-Way ANOVA was run.

### Instruments

The study data were collected using a demographic information form and AGQ. The demographic information form included age, education level, marital status, and type and duration of substance abuse. Indeed, AGQ assessed the patients’ level of aggression. This questionnaire was invented by Buss and Perry in 1992 [12]. In Iran, the psychometric characteristics of AGQ were determined by Najarian. Accordingly, its test-retest reliability coefficients were reported to be 64 and 74% [13].

## Results

This study was conducted on 36 participants. Most of the participants were between 20 and 40 years old and a few of them were less than 20 years old. The mean age of the participants was 33 and 31 years in the intervention and control groups, respectively. Besides, 66% of the participants were single and 33% were married. Additionally, 50, 38, and 11% of the participants had below diploma degrees, diplomas, and B.Sc. degrees, respectively. Moreover, 52, 22 and 25% of the participants abused opium, heroin, and methadone, respectively (Tables 1 and 2).

**Table 1:** Comparison of qualitative demographic characteristics among participants in control and experiment group

*P*-value	two groups	control group		Experiment group		Variable	
	% Of total	Percent	Number	Percent	Number		
0.52	66.6	72.2	13	61.1	11	Single	Marriage status
	33.2	27.7	5	38.8	7	Married	
0.28	49.9	55.5	10	44.4	8	High school diploma	Education
	38.8	27.7	5	50.0	9	Diploma	
	11	16.6	3	5.50	1	B.S	
	0.00	0.00	0	0.00	0	M.S	
0.87	52.7	50.0	9	55.5	10	Opium	Opioid type
	22.2	22.2	4	22.2	4	Heroin	
	24.9	27.7	5	22.2	4	Methadone

**Table 2:** Comparison of quantitative demographic characteristics of participants in the experiment and control groups

*P*-value	control group		Experiment group			Variable
	Percent	Number	Percent	Number		
0.608	16.6	3	22.2	4	Less than 20 years	Age
	61.1	11	50	9	20 to 40 years	
	22.2	4	27.7	5	Above 40 years	
	31.56		33.22		mean	
	9		9		Standard deviation	

The intervention group’s mean level of aggression was 54.11 before the intervention and 47.72 after that. These measures were respectively 59.17 and 63.72 in the control group. Based on the results of paired t-test, the level of aggression changed significantly in both groups after the intervention.

According to Table 3, the mean of aggression level decreased by seven scores in the intervention group, but increased by four scores in the control group, which were both statistically significant. In the intervention group, the mean of aggression level decreased by at least one score and at most 11 scores. On the other hand, this measure increased by at least one score and at most eight scores in the control group.

**Table 3:** Comparison of the mean aggression level before and after the intervention between the experiment and control groups

*p*-value	Control group		Experiment group		
	Standard deviation	Mean	Standard deviation	Mean	
0.188	12	59.17	10.58	54.11	Before intervention
0.001	11	63.72	10.00	47.72	After intervention
–	0.013		0.015		P-value

## Discussion

Anger and aggression are not only socially unacceptable, but they are also considered to be the risk factors for health problems. Moreover, the risk of showing aggressive and dangerous behaviors was higher among individuals with mental illnesses [14]. The present study aimed to assess the impact of group education based on anger management on aggression level among patients suffering from substance abuse. This is one of rather few studies of anger management with adults, though. In the current study, 50, 38, and 11% of the participants had below diploma degrees, diplomas, and B.Sc. degrees, respectively. Therefore, most of the participants had below diploma degrees, which is in agreement with the results of some studies [15, 16], but inconsistent with some others [17]. Low level of education is a predisposing factor for dependency on substances. However, few studies have been conducted on the prevalence of substance abuse among individuals with below diploma degrees. Thus, it is not clear whether the large number of dependent cases is related to the higher availability of substances or the openness of this group in expressing their dependency compared to those with high education levels.

In the current study, the results of data analysis indicated that the level of aggression was higher than the average level before the intervention. This is compatible with the results of some studies [3, 18], but not consistent with some others [19]. Increase of aggression among patients and other society members can be attributed to high levels of stress in urban and industrial communities. Yet, our study findings revealed that interventions on anger management skills could significantly decrease the level of aggression among patients dependent on opioid materials. While the control group did not receive the training, the level of aggression was higher suggesting that physical and recreational environments in mental hospitals may be vital to reduce the influence of the environment on patients’ aggression.

Having the skill of anger management will increase one’s adaptive and psychological capacity. Lack of awareness about the right ways to live and absence of necessary skills, provide the context for mental illness and social dilemmas, which are mainly the result of poor education. Anger management training can raise people’s awareness about the concept of anger, the factors causing violence and ways to control it. Moreover, it can promote healthy and useful social behaviors and how to deal with psychological stress in order to enable people not to use aggressive behaviors in their interactions with others.

This is compatible with the results of some previous investigations [20, 21]. However, several studies assessing the effect of cognitive behavioral approach on anger management came to contradictory results. For instance, Ozabaci conducted a meta-analysis in 2011 and found that this approach was not as effective as expected in aggression among children and adolescents [22]. Farajzadeh also employed the same approach in group education for anger management. They evaluated the effect of this approach on aggression and social qualification of adolescents living in Welfare Organization’s dormitories in Tabriz, Iran. They found that the intervention was not effective in changing adolescents’ social skills and aggression levels [23]. The difference among the results might be due to differences in contents and durations of educational interventions. Also this difference may also be due to the difference in context and nurture of the dormitories’ welfare organization.

## Conclusions

The results of this study showed that anger management education could decrease the level of aggression and develop health promotion among patients abusing substances. Considering the importance of anger management and aggression control, anger management education performed by nurses and other healthcare members is highly crucial. Moreover, the level of aggression was closely related to substance abuse. Thus, it is a main barrier against quitting addiction. Therefore, it is essential to encourage nurses to establish permanent educational programs, especially in psychiatric wards, for controlling patients’ anger through anger management using group education.
